# A Systematic Review of Autistic People and the Criminal Justice System: An Update of King and Murphy (2014)

**DOI:** 10.1007/s10803-022-05590-3

**Published:** 2022-05-30

**Authors:** J. Collins, K. Horton, E. Gale-St. Ives, G. Murphy, M. Barnoux

**Affiliations:** Tizard Centre, University of Kent, Canterbury, CT2 7NF UK

**Keywords:** Autism, ASD, Crime, Offending, Criminal justice

## Abstract

The purpose of this paper was to determine whether recommendations made by King & Murphy (Journal of Autism and Developmental Disorders 44:2717–2733, 2014) in their review of the evidence on autistic people in contact with the criminal justice system (CJS) have been addressed. Research published since 2013 was systematically examined and synthesised. The quality of 47 papers was assessed using the Mixed Methods Appraisal Tool. Findings suggest a limited amount of good quality research has been conducted that has focused on improving our understanding of autistic people in contact with the CJS since 2013. Methodological limitations make direct comparisons between autistic and non-autistic offenders difficult. Autistic people commit a range of crimes and appear to have unique characteristics that warrant further exploration (i.e., vulnerabilities, motivations for offending).

## Autism and the Criminal Justice System

The prevalence of autism has been much debated over the years, but it was estimated at approximately 1 in a 100 in England by Brugha et al. (2011). However, a recent review has suggested that the prevalence varies across the world (Chiarotti & Venerosi, 2020), with a recent study in Poland, which suggested that 5.29/1000 of children aged 0–16 years were autistic (Skonieczna-Zydecka et al., 2017) and in Spain a prevalence of 11.8/1000 autistic children aged 6–10 years was reported (Pérez-Crespo et al., 2019). It is now accepted that autistic individuals have varying degrees of strengths and challenges in relation to social communication, social interaction, and social imagination (Wing & Gould, 1979), the spectrum being considered a continuum. Following screening and a developmental interview (such as the Autism Diagnostic Interview-Revised; Rutter et al., 2003), as well as an observation of the person themselves engaging in specified tasks (using a measure such as the Autism Diagnostic Observation Schedule, Lord et al., 2000), a diagnosis of Autism can be made by a qualified clinician using the criteria from the International Classification of Diseases-10 or 11 (World Health Organization, 1992, 2019), and the Diagnostic Statistical Manual of Mental Health Disorders, 5th Edition (DSM-5; American Psychiatric Association, 2013).

Previous research has suggested autistic adults are over-represented in some parts of the Criminal Justice System (CJS), with some evidence indicating autistic individuals are more likely to have contact with the police than the general population (Lindblad & Lainpelto, 2011; Siponmaa et al., 2001). However, estimates of the prevalence of autism within the CJS have varied considerably over the years, no doubt partly due to different methodological approaches within the research. Arguably, according to some studies, autistic people are no more or less likely to come into contact with the CJS compared to non-autistic people (Brookman-Frazee et al., 2009; Cheely et al., 2012; Hippler et al., 2010; Mourisden et al., 2008; Woodbury-Smith et al., 2006). Where criminal offending does occur, research has suggested autistic people engage in a variety of offence types, including violence (e.g., Barry-Walsh & Mullen 2004; Cheely et al., 2012), arson (Woodbury-Smith et al., 2010), sexual offending (e.g., Mouridsen et al., 2008; Långström et al., 2008; Søndenaa et al., 2014), and stalking (e.g., Stokes & Newton, 2004; Stokes et al., 2007).

King & Murphy (2014) sought to explore these issues further by conducting a systematic review of the evidence pertaining to autistic people within the CJS, which focused on summarising research conducted up to January 2013. The authors explored research on the prevalence of autism amongst people in contact with the CJS (at various stages in it), the occurrence and types of offending, and the presence of co-occurring psychiatric conditions. Findings suggested autistic people were less likely to offend than other people of the same age and gender. However, the evidence on types of crime was inconsistent, perhaps due to the poor quality of research methodology, though it seemed that ‘offences against the person’ were more common than property related offences (King & Murphy, 2014). There did also appear to be a trend of higher rates of psychosis and personality disorder diagnoses, rather than other mental health diagnoses, among autistic people in the CJS, although the samples in the studies were biased towards service-users recruited from mental health settings. The factors identified in the literature which might predispose autistic people to offend included social naiveté, misunderstanding of social situations, lack of understanding of the rules, and obsessional interests (King & Murphy, 2014).

King & Murphy (2014) highlighted the limitations of the evidence base and consequently made several recommendations including the need for consistency in the methods used to diagnose autism, and the need for further research into the experiences of autistic people who encounter the CJS. Additionally, King and Murphy argued that few researchers used representative samples, and even when they were representative, the participants came from within specific geographical locations. Only a small number of studies used comparison groups and there were differences in how offending behaviour was defined. In addition, King & Murphy (2014) identified that the lack of research on autistic female offenders was problematic and concluded that the study of the relationship between autism and offending was in its infancy.

## Recent Developments in Policy

Since the publication of King and Murphy’s (2014) review, recognition of autism has increased with new law, policy and guidance having been introduced in England and Wales, including the Care Act (2014), ‘Transforming Care for people with learning disabilities—next steps’ (NHS England, 2015b), ‘Building the Right Support’ (NHS England, 2015a) and ‘Autism Spectrum Disorder in Adults’ (NICE, 2016). Further research on supporting autistic prisoners aimed to develop a set of Autism Accreditation standards within prisons (Turner et al., 2016). And the Liaison and Diversion Services (NHS England & NHS Improvement, 2019) has been gradually introduced to support vulnerable individuals who encounter the CJS (following recommendations in the Bradley report, 2009). In addition, the Police and Criminal Evidence Act Code of Practice was revised in 2019 to provide further guidance to officers when detaining, cautioning, and interviewing vulnerable suspects, including autistic people (Home Office, 2018). However, ‘Beyond the High Fence’ (NHS England, 2019) suggested the understanding around autism remained poor in forensic services, underpinned by insufficiently experienced staff, who were unable to adequately support autistic people. Reports of high levels of physical and medical restraint of autistic people were confirmed in a review of restraint, seclusion and segregation conducted by the Care Quality Commission (2020) which found that the majority (64%) of the 66 cases they investigated had autism.

Similar policy developments have been seen beyond the UK with the introduction of the Autism CARES Act (2019) in the USA and the National Call to Action to improve services issued by the Australian Advisory Board on Autism Spectrum Disorder in 2011. However, in other countries across the world there may well be even more of a need for an improved understanding of autism, with few policies and guidelines in existence.

Whilst new legislation, policies, and guidance have been introduced since King and Murphy’s (2014) review, particularly within the UK, the question remains as to whether our research knowledge and understanding of autistic individuals who encounter the CJS has progressed, whether sufficient changes have been implemented and evaluated to improve practice, and whether the experiences of autistic adults who encounter the CJS has improved.

### Aims

The current review therefore aims to provide an update of King and Murphy’s (2014) paper to firstly identify whether the gaps in the research and their recommendations have been addressed, as well as to identify whether our knowledge and understanding of autistic people who encounter the CJS has improved. In reviewing literature published since the original review, authors aimed to determine a more accurate estimate of prevalence of autism in the CJS, and outline the characteristics associated with autism and offending behaviour.

## Method

### Design

A mixed methods systematic review of the research on autistic adults with a history of criminal offending was conducted. To provide continuity and for comparisons to be made between King & Murphy (2014) and the current update, previous search methods were duplicated.

Database and ancestry searches resulted in 47 articles that met the specific inclusion criteria. Data was extracted and a quality assessment was conducted using the Mixed Methods Appraisal Tool (Hong et al., 2018, 2019). There are five criteria for appraising studies and ratings vary from 0* (none of the criteria are met) to 5* (all criteria are met). A data extraction template was used to record relevant information under the following heading: Title, author, year of publication, study population, number of participants, methods, data on prevalence of autism/offending, types of offending behaviour, psychiatric diagnosis, and other information. The quantitative and qualitative findings were then summarised based on the previous review conducted by King & Murphy (2014), under the following headings:

- Prevalence of autism in offender populations.
- Prevalence of offending behaviour in autistic people.
- Types of offence committed by autistic people.
- Co-morbid psychiatric diagnoses.
- Other results.

### Search Strategy

A systematic search of the literature was conducted on 10th December 2018 by the second author and updated on 1st April 2021 by the first author of the same electronic databases (PsychINFO, Medline, Criminal Justice Abstracts, Cochrane Database of Systematic Reviews) as searched by King & Murphy (2014), except for the National Autistic Society database due to its closure post 2014. In addition, a further two databases were added to broaden the search (PsychARTICLES, CINAHL Plus with Full Text) using identical search terms to find articles relating to autism and offending. Date limiters were applied to include papers from 1st January 2013 to 1st April 2021. To ensure continuity, the same search terms were used to find articles and these are provided in Table 1.

**Table 1:** Search terms

Autism terms	Offending terms
Autis*	“Criminal Justice System”
ASD	Prison
ASC	Probation
Asperger*	Court
“Pervasive developmental disorder”	Secure
	Forensic
	Crim*
	Offen*

*Used to search for any word that includes the letters before the asterisk

### Eligibility Criteria

The inclusion and exclusion criteria applied in this review matched the King & Murphy (2014) systematic review and aimed to consolidate and evaluate current research on autism and offending. Articles were reviewed to ensure that they met the following inclusion criteria:

- Written in English.
- Published in peer reviewed journals.
- Included participants with an autism diagnosis according to ICD-10/ICD-11 or DSM-IV-TR/DSM-5.
- Participants with involvement in the CJS (those who were suspects, offenders and in contact with the CJS including contact with police, courts, probation, and prison services in addition to forensic services such as secure hospitals).

Articles were excluded for the following reasons:

- Individuals with only apparent ‘autistic symptoms’ and no attempt to diagnose.
- Studies that included witnesses or victims of crime, rather than suspects or offenders.
- Single case studies, dissertations, and review papers.
- Articles which focused on treatment.

### Identification of Studies

**Fig. 1:**
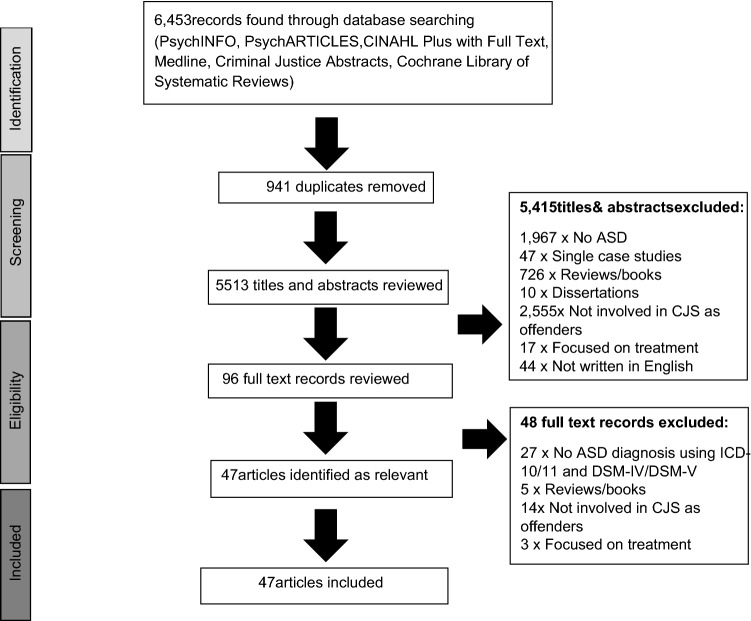
PRISMA chart of search results

The Preferred Reporting Items for Systematic Reviews and Meta-analyses (PRISMA; Knobloch et al., 2011) was used to identify relevant studies (Fig. 1). The initial search yielded 6453 papers. After duplicates were removed, the titles and abstracts of 5512 papers were screened by the first author and 5416 papers were excluded based on the above inclusion and exclusion criteria. Therefore, the full texts of 96 papers were identified by the first author for review. Following a further review of the 96 articles against the eligibility criteria, 47 articles were included.

### Data Extraction

The 47 studies were recorded, and relevant data such as author, year of publication, sample, methods, data on prevalence, types of offending behaviour, psychiatric diagnosis was extracted (see Table 2). A total of 36 publications were either cohort studies, cross-sectional studies, case control studies or descriptive studies. A total of six studies used qualitative designs and included interviews and case studies. Lastly, five studies used a mixed methods design, which included surveys and interviews.

**Table 2:** Included studies

Author, Year, Country & Title	Study population	Number of participants	Methods	Outcomes	MMAT Rating
1. Alexander et al. (2015) (UK). Arson or firesetting in offenders with intellectual disability: Clinical characteristics, forensic histories, and treatment outcomes.	Adults with intellectual disability treated in a specialized forensic inpatient service in England for people with mild intellectual disability and offending behaviour over a 6-year period.	30 Adults with intellectual disabilities and a history of firesetting (22 males, 8 females). Age: M = 29.23, SD = 9.19 105 adults with intellectual disabilities and no history of firesetting (85 males, 20 females) Age: M = 30.69, SD = 9.27	Quantitative between groups design. Retrospective file review conducted to collect data. The service used the ICD-10 to make diagnosis.	Prevalence 34.4% of adults with intellectual disabilities were autistic (n = 46). **Offence ** Firesetting.	2*
2. Billstedt et al. (2017) (Sweden). Neurodevelopmental disorders in young violent offenders: Overlap and background characteristics.	Young male sentenced for violent offences (18–25 years) who served time between March 2010 and July 2012 at any of 9 prison facilities in Western region of the Swedish Prison and Probation Service. Same sample used by Hofvander et al. (2019) and Widinghoff et al. (2019).	269 Male violent offenders with neurodevelopmental disorders Age: 18–25 years (M = 22.3, SD = 1.9) 11% of the offenders had ASD (n = 30).	Quantitative cohort study using retrospective data. Assessment of autism involved a clinical assessment using DSM-IV (APA, 2000) and the Autism Spectrum Quotient (Baron-Cohen et al., 2001).	Prevalence 11% of the offenders were autistic (n = 30). Offence Violence Psychopathology ADHD, Tourette syndrome, early onset conduct disorder.	3*
3. Buitelaar & Ferdinand (2016) (Netherlands). ADHD undetected in criminal adults.	Adults with ADHD recruited from a multicentre forensic outpatient clinic in the Netherlands for people with a psychiatric disorder.	106 Male adult outpatients of a clinic for forensic mental health care suspected of having ADHD. Age: 18–51 years (M = 29.4, SD = 9.1)	Quantitative cohort study. Diagnosis of autism made using psychiatric assessment and DSM-IV (APA, 2000).	Prevalence 0.94% diagnosed with pervasive developmental disorder (n = 1).	5*
4. Esan et al. (2015) (UK). The clinical, forensic, and treatment outcome factors of patients with autism spectrum disorder treated in a forensic intellectual disability service.	Service users in a specialised forensic inpatient intellectual disability hospital in England.	138 Patients treated over a period of 6 years. Age: 29+ (M = 30.14).	Quantitative between groups design using a retrospective case file review. Diagnosis made using ICD-10.	Prevalence 30.4% autistic patients (n = 42; 36 males & 6 females). Offence Violence, sexual, and arson. Psychopathology Psychosis, bipolar disorders, depressive disorders, harmful use substances, personality disorder. Other characteristics Victim of abuse (including sexual abuse), self-harm, verbal aggression.	2*
5. Glover & Brown (2015) (UK). People with intellectual disabilities hospitalised by courts in England.	Data taken from the national consensus of psychiatric inpatients with intellectual disabilities and/or autism.	3,250 psychiatric inpatients with intellectual disabilities and/or autism from 104 hospital provider organisations. 1,017 (42%) of detained patients were under part III of MHA (2007). In all but two cases the individual was convicted of an imprisonable offence (87% male).	Quantitative descriptive cohort study Diagnosis data taken from the first National Consensus of psychiatric inpatients with intellectual disabilities.	Prevalence 17% had intellectual disability and were autistic. 6% were autistic.	4*
6. Griffiths et al. (2018) (UK). Seclusion: the association with diagnosis, gender, length of stay and HoNOS-secure in low and medium secure inpatient mental health service.	Service users admitted to low & medium secure units across four regional sites in the UK between 2007 and 2015.	347 service users (88% males & 12% females). Age: 18 + (M = 32.5).	Quantitative cohort study using retrospective data. Diagnosis made using ICD-10.	Prevalence 14% autistic.	4*
7. Helverschou et al. (2015) (Norway). Offending profiles of individuals with autism spectrum disorder: A study of all individuals with autism spectrum disorder examined by the forensic psychiatric service in Norway between 2000 and 2010.	Individual offenders who have undergone forensic examination between 2000 and 2010 in Norway. Same total population sample as Søndenaa et al. (2014).	Total population: 3,382 offenders. Study sample: 41 males & 7 females. Age: (M = 28.3, SD = 11.3)	Quantitative cohort study using retrospective data. A forensic examination was conducted using ICD-10 or previous mental health services.	Prevalence 14.2% autistic. Offence Violence, sexual offences, vandalism including arson, theft/robbery, and fraud. Psychopathology Intellectual disability, drug related disorder, ADHD, personality disorder, psychosis, affective disorders, obsessive compulsive disorder, adjustment disturbances, tourette syndrome, phobia, somatoform disorders, pyromania, fetishism, paedophilia, behavioural disturbances Other characteristics History of foster/institutional care. Motivations included social misunderstanding, idiosyncratic rationalisations, special interests, social naivete, revenge. Educational attainments poor. Employment status low. Limited social networks.	3*
8. Hill et al. (2019) (Portugal). Characteristics and personality profiles of first 100 patients admitted to a secure forensic adolescent hospital.	First 100 patients admitted to a mixed gender adolescent medium secure forensic hospital between 2008 and 2013.	100 adolescents (61% male & 39% female) Age: 12–18 (M = 16.59) Ethnicity: 82% White British, 18% mixed race, Asian, or Black British.	Quantitative cross-sectional study using retrospective data. Diagnosis made using ICD-10.	Prevalence 7 adolescents (6 males & 1 female) with pervasive developmental disorder.	4*
9. Hofvander et al. (2019) (Sweden) Few differences in the externalizing and criminal history of young violent offenders with and without autism spectrum disorders.	Young violent autistic offenders and offenders without autism recruited from the Development of Aggressive Antisocial Behaviour Study, a nationally representative cohort of all young adult male offenders (aged 18–25 years) convicted of hands on violent (including sexual) offences and imprisoned in one out of nine prisons (low to high secure) in the Western region of the Swedish Prison and Probation Service between March 2010 and July 2012. Same sample used by Billstedt et al. (2017) and Widinghoff et al. (2019).	269 violent male offenders.	Quantitative cohort study using retrospective data. Assessment of autism included the DSM-IV (APA, 2000), the Asperger Syndrome/high functioning autism Diagnostic Interview (ASDI Gillberg et al., 2001), a collateral interview (the Autism-Tics, ADHD and other Comorbidities inventory/A-TAC; Hansson et al., 2005), Diagnostic Interview for Social & Communication disorders” (DISCO; Wing et al., 2002), with parents/caregivers or an “Autism Diagnostic Observation Schedule” (ADOS; Lord et al., 2000).	Prevalence 9.7% autistic (n = 26). Offence Sexual offending. Psychopathology ADHD, antisocial personality disorder, substance use disorder. Other characteristics/risk factors Offending history (violence, sexual, drug offences, property crimes, traffic crimes, fraud, externalizing and antisocial behaviours, placements in foster homes. Psychopathy assessed and no significant differences between autistic and non-autistic group.	4*
10. Lindsay et al. (2014) (UK). A comparison of referrals with and without autism spectrum disorder to forensic intellectual disability services.	Referrals made to forensic community and secure intellectual disability services in one calendar year in England and Scotland. Study sites comprised 24 separate services.	477 referrals made to forensic intellectual disability services. Age: 18 + years	Quantitative between group design using a file review. Previous diagnosis of autism.	Prevalence 10% autistic (n = 48). Offence Physical aggression, verbal aggression, stalking, contact sexual offence, non-contact sexual, property damage, fire starting, theft, substance misuse. Psychopathology Intellectual disability Other characteristics History of abuse, birth defects.	4*
11. Murphy et al. (2017) (UK). Incompatibilities and seclusion of patients with an autism spectrum disorder detained in high-secure psychiatric care.	Autistic adults detained in a high secure psychiatric care in the UK.	Total population: 198 Sample: 8 male autistic individuals detained in high secure psychiatric care	Quantitative cohort study. Diagnostic assessment included Autism Diagnostic Interview Revised (Lord et al., 1994) with a caregiver where possible, completion of an Autism Diagnostic Observation Schedule 2 (Lord et al., 2012), completion of the Adult Asperger Assessment (Baron-Cohen et al., 2005).	Prevalence 4% autistic. Psychopathology ADHD, personality disorder (notably antisocial) and psychosis Other characteristics Incompatibility with other patient, episode of seclusion due to behaviour and risk.	4*
12. Newman et al. (2015). (Australia). A hermeneutic phenomenological examination of the lived experience of incarceration for those with autism.	Offenders in seven prisons (low, medium, and high secure) in New South Wales, Australia.	8 male offenders with ASD in prison of 20 potential participants identified (prison population was 10,000). Age: 21–43 years (M = 24)	Qualitative interviews. DSM-IV (APA, 2000) used to diagnose autism.	Prevalence 0.2% autistic. Other characteristics Anxiety, victim of abuse	5*
13. Sheridan & Pyszora ( 2018). (Australia). Fixations on the police: An exploratory analysis.	Persons who were known to the State Security Investigation Group of the Western Australia Police as they were fixated on the police.	30 people with recorded history of fixating behaviour towards the police (76.7% men). Age: 18–75 (M = 45.72, SD = 14.72).	Quantitative descriptive study using case file review. Evidence of diagnosis in police file.	Prevalence 3.3% Autistic.	5*
14. Slaughter et al. ( 2019) (USA) Criminal behaviour and school discipline in juvenile justice-involved youth with autism.	Juvenile justice involved youth (n = 58,678) with and without autism with learning disabilities and other special educational needs identified from criminal case records of juvenile justice-involved youth between 2006 and 2012 in Connecticut.	Autistic juveniles involved in the CJS (n = 143) Non-autistic, with learning disabilities and other special educational needs involved in the CJS (n = 572)	Quantitative between groups design using secondary data. Diagnosis taken from the autism primary educational classification - using the IDEA (2004), a federal law that ensures services for students with disabilities, so they have access to public education.	Prevalence 0.24% autistic. Offence Crimes against persons, status offences, property violations, drug law violations, public order offences. Other characteristics/risk factors School discipline incidents reported.	4*
15. Søndenaa et al. (2014) (Norway). Violence and sexual offending behaviour in people with autism spectrum disorder who have undergone a psychiatric forensic examination.	Forensic examination reports over a 10-year period in Norway between 2001 and 2011 where the charged person was diagnosed with ASD and either a violent or sexual offence. Same population sample as Helverschou et al. (2015).	Total number of cases: 3382 Violent offence (n = 21) Sexual offence (n = 12) 28 males & 5 females Age: 15–54 years (M = 25.5)	Quantitative cohort study using a retrospective file review. Diagnosis made using ICD-10.	Prevalence In 1.4% of cases the offender was autistic. Offence Sexual offending, violence, fraud, theft, property damage, arson, illegally calling 911, traffic crimes or drug crimes. Psychopathology Psychotic disorder, affective disorder, personality disorder, intellectual disability, other developmental disorders, substance abuse problems. Other characteristics Mean age for diagnosis was 23.2 years (SD = 11.3). Motivated by revenge, misunderstanding/ idiosyncratic ideas, special interests.	4*
16. Sturup (2018). (Sweden). Comparing serial homicides to single homicides: A study of prevalence, offender, and offence characteristics in Sweden.	Convicted homicide offenders in Sweden between 1973 and 2012 and between 2007 and 2009.	25 serial homicide offenders (84% males & 16% females) 201 single homicide offenders (93% males & 7% females) Age: (M = 29 years)	Quantitative longitudinal study with a between groups design. Diagnosis made using DSM-IV (APA, 2000) by the multidisciplinary team.	Prevalence Serial cases: 33% were autistic (n = 8). Single cases: 4% were autistic (n = 5). Offence Murder, infanticide, and manslaughter. Psychopathology Personality disorder	4*
17. Sutton et al. (2013) (USA). Identifying individuals with autism in a state facility for adolescents adjudicated as sexual offenders: A pilot study.	Adolescents in a secure state facility for sexual offenders in the USA.	37 males sentenced for sexual offending. Age: 14–20 years (M = 17) Ethnicity: 59.5% Caucasian, (n = 22), 35.1% African American (n = 13), and 5.4% Hispanic (n = 2).	Quantitative between groups design. Assessment included the Asperger’s Syndrome Diagnostic Scale (Myles et al., 2001) and the DSM-IV (APA, 2000).	Prevalence 59.55% with autism (n = 22). Offence Sexual offending and a variety of criminal acts. Psychopathology Negative mood/low levels of enjoyment and use of alcohol.	4*
18. van den Bogaard et al. (2013) (Netherlands). Comparison of intellectually disabled offenders with a combined history of sexual offenses and other offenses versus intellectually disabled offenders without a history of sexual offenses on dynamic client and environmental factors.	Sex offenders in a residential treatment facility specialised in people with intellectual disability and additional mental health problem in the southeast of the Netherlands.	Total sample: 69 males with mild intellectual disability or borderline intellectual functioning. 30 with a history of combined sex and other types of offences 30 with a history of non-sexual offences.	Quantitative between groups design. Diagnosed using DSM-IV-TR (APA, 2000).	Prevalence 28 were diagnosed with pervasive developmental disorder (19.9%): Mixed sex offenders (n = 15) Non sex offenders (n = 13) Offence Sexual offending.	3*
19. Walters et al. (2013) (USA). Maltreatment and depression in adolescent sexual offenders with an autism spectrum disorder.	Adolescents adjudicated delinquent due to a sexual offence in a state residential facility in Pennsylvania.	43 Male adolescent offenders Age: 15–20 years (M = 17.9) Ethnicity: 54% Caucasian, 35% African American, 10% Hispanic	Quantitative cohort study. Assessment of autism using DSM-IV-TR (APA, 2000).	Prevalence 62.8% of adolescent offenders diagnosed with autism (n = 27). Offence Sexual offending Psychopathology Depressive symptoms. Other characteristics/risk factors Emotional abuse and neglect	4*
20. Widinghoff et al. (2019) (Sweden). Gambling disorder in male violent offenders in the prison system: Psychiatric and substance‑related comorbidity.	Young violent autistic offenders and offenders without autism recruited from the Development of Aggressive Antisocial Behaviour Study, a nationally representative cohort of all young adult male offenders (aged 18–25 years) convicted of hands on violent (including sexual) offences and imprisoned in one out of nine prisons (low to high secure) in the Western region of the Swedish Prison and Probation Service between March 2010 and July 2012. Same sample used by Billstedt et al. (2017) and Hofvander et al. (2019).	264 Male violent offenders Age: 18–25 years (M = 22.3).	Quantitative between groups design. Assessments included the DSM-IV (APA, 2000), the Asperger Syndrome/high functioning autism Diagnostic Interview (ASDI; Gillberg et al., 2001), a collateral interview (the Autism-Tics, ADHD and other Comorbidities inventory/A-TAC; Hansson et al., 2005), Diagnostic Interview for Social & Communication disorders” (DISCO; Wing et al., 2002), with parents/caregivers or an “Autism Diagnostic Observation Schedule” (ADOS; Lord et al., 2000).	Prevalence 9.5% diagnosed with autism (n = 25). Offence Violent and sexual offending. Psychopathology None of those diagnosed with autism had a gambling disorder.	4*
21. van Wijk et al. (2018) (Netherlands). Animal abuse: Offender and offence characteristics. A descriptive study.	Offenders convicted of abusing animals. Data collected from the National Police, the National Inspectorate Animal Protection, the national reporting station, and the Dutch Probation Service.	97 Adults convicted of animal abuse (86 males & 11 females).	Quantitative descriptive study using retrospective file reviews. Diagnosed using DSM-IV (APA, 2000).	Prevalence 6 Participants had pervasive developmental disorder (6.2%). Offence Animal abuse Psychopathology Behavioural disorder and suspicion of sexual sadism (n = 1)	4*
22. Williams et al., (2020) (UK). Learning disability, autism, and the Criminal Procedure (Scotland) Act.	Active Criminal Procedure (Scotland) Act orders in January 2018.	All CPSA orders active on the 3 January 2018 were identified (n = 580). Those with and without learning disabilities compared for length of detention.	Quantitative between groups study. Possible/definite diagnosis of Autism.	Prevalence 4.7% had possible/ definite diagnosis of Autism (n = 27). Psychopathology Intellectual disability, mental illness, and personality disorder	3*
23. Young et al. (2018) (UK). Neurodevelopmental disorders in prison inmates: Comorbidity and combined associations with psychiatric symptoms and behavioural disturbance.	Male prisoners (sentenced or on remand) in Scotland UK between 2011 and 2013.	390 Male prisoners Age: 18–50 years (M = 30.3, SD = 8.35)	Quantitative cross-sectional design. Assessment of autism included the use of the Autism Quotient (Baron-Cohen et al., 2001).	Prevalence Prevalence of autism was 9%. Psychopathology Intellectual disability, ADHD. Other characteristics Higher scores on the machismo subscale of the Disruptive Behavioural Social Problems (Young et al., 2018), and the behavioural disturbance and attitudes towards violence sections of the Maudsley Violence Questionnaire (Walker, 2005).	4*
24. Yu et al. (2021) (USA). Young adults with autism spectrum disorder and the criminal justice system.	Young adults recruited from the juvenile justice system and adult criminal justice system for young adults aged 17–23 years with ASD.	Total sample: 4850 Comprising three participant groups: autistic adults (n = 606), intellectual disabilities (n = 1271), and population control group (n = 2973).	Quantitative with matched control group longitudinal study. Diagnosis made using DSM-IV-TR (APA, 2000).	Prevalence 12.5% autistic adults identified (n = 606). Offence Offenses against public order (55%), crimes against property (55%), and crimes against person (40%). Other characteristics History of offending.	4*
25. Heeramun et al. (2017) (Sweden). Autism and convictions for violent crimes: Population-based cohort study in Sweden.	Stockholm youth cohort- a total population-based record of children aged 0–17 years who were resident in Stockholm County between 2001 and 2011, with a diagnosis of ASD, also on the Swedish national crime register containing all convictions in Sweden since 1973.	295,734 individuals recorded on the Stockholm youth cohort (5739 had a recorded ASD diagnosis) Resident between 2001 and 2011 Age: 15–27 years	Quantitative between groups design Diagnosis made using the ICD-9 & 10, and DSM-IV (APA, 2000).	Prevalence 4.4% of those who were autistic were convicted of at least one violent offence (n = 250). Offence Violence Psychopathology Intellectual disabilities, psychotic disorder, drug and alcohol misuse, personality disorder, ADHD, Conduct Disorder. Other characteristics A range of parental and familial characteristics and a later age at first recorded diagnosis of autism.	5*
26. Rava et al. (2017) (USA). The prevalence and correlates of involvement in the criminal justice system among a nationally representative sample of youth with autism.	Used secondary data from National Longitudinal Transition study, which was a nationally representative study of youth enrolled in special education through Local Education Agencies and state supported special schools.	11,270 enrolled between 2001 and 2009, including 920 with autism (83.1% male, 16.9% female) Age: 15–17 years. Ethnicity: white (67.2%), African American (21.4%), Multi/other (11.5%), Hispanic (10%).	Quantitative descriptive collecting data from education files, telephone interviews and mail surveys. Diagnosed using secondary data from the National Longitudinal Transition study.	Prevalence By age 21, 20% of autistic youth had been stopped and questioned by the police, and 5% had been arrested. Psychopathology ADHD Other characteristics Victimisation and social isolation.	3*
27. Salerno & Schuller (2019) (Canada). A mixed-methods study of police experiences of adults with autism spectrum disorder in Canada.	Autistic adults living in Canada. Same sample used by Salerno & Schuller in 2019 and 2020.	35 autistic adults (21 women and 13 men, 1 unknown) who had and had not encountered the CJS. Ethnicity: 64% White European, Age: 18–65 (M = 36.9, SD = 11.96)	Mixed methods survey. Self-report a diagnosis of autism, pervasive developmental disorder, or Asperger’s syndrome.	Prevalence 5.7% of those who were autistic reported being convicted (n = 2). Psychopathology Anxiety and depression. All participant described as very mildly impaired. Other characteristics History of police contact and victimisation.	4*
28. Salerno-Ferraro & Schuller (2020) (Canada). Perspectives from the ASD community on police interactions: Challenges & recommendations.					4*
29. Tint et al. ( 2017) (Canada). Correlates of police involvement among adolescents and adults with autism spectrum disorder.	Parent report of autistic adolescents and autistic adults in Ontario, Canada.	284 autistic adolescents and adults. Information gathered over 12–18 months (78.5% male). Age: 12–56 (Mdn = 17, SD = 6.16).	Quantitative longitudinal cohort study using a survey. Formally diagnosed with autism verified by meeting a cut-off score of 12 or more on the Social Communication Questionnaire (SCQ; Rutter et al., 2003).	Prevalence Criminal charges were bought against two autistic people (0.7%). Offence Physical and verbal aggression. Psychopathology Intellectual disabilities, psychiatric diagnosis Other characteristics Histories of aggression	3*
30. Bosch et al. (2020) (Netherlands). Inpatient aggression in forensic psychiatric patients with autism spectrum disorder: The role of risk and protective factors.	Service users with ASD admitted to a Dutch medium secure forensic psychiatric hospital.	32 service users with ASD and a criminal charge admitted to a medium secure forensic psychiatric hospital. 90.6% male, n = 29). Age: 22.4–57.3 years (M = 37.7, SD = 11).	Quantitative naturalistic prospective cohort study ASD diagnosis assessed according to DSM-IV criteria and diagnosed by a registered psychologist/ psychiatrist.	Offence 75% of the sample was previously convicted for at least one violent crime. 46.9% was previously convicted for property crime and 21.9% was previously convicted for sexual offenses. Psychopathology Comorbid psychiatric disorders, including substance-related disorders, schizophrenia and other psychotic disorders, paraphilic disorders, personality disorders, intellectual disability and ADHD.	3*
31. Gibbs & Haas (2020) (Australia). Interactions between the police and the autistic community in Australia: Experiences and perspectives of autistic adults and parents/carers.	Autistic people who had interacted with police in Australia over a period of 5 years.	50 autistic adults Male (n = 17, 34%), female (n = 28, 56%), other (n = 4, 8%), unknown (n = 1, 2%) Age: 18–64 Details on 65 autistic people as reported by their parent/carers Male (n = 54, 83.07%), female (n = 10, 16.3%), other (n = 1, 1.7%) Age: 61.5% were 17 or younger (n = 40).	Mixed methods: Survey and interview with sub-sample of 12 autistic adults (7 females, 5 males), and 18 parent/carers of 1 female and 17 males. Adults who self-reported having a professional diagnosis of any form of autism, or parents/carers of a person of any age with the diagnosis of autism.	Offence Drug offences, violence/physical assault, sexual offences, traffic incident/driving offence, domestic violence. Psychopathology Intellectual disability, comorbid mental health, current/historical substance abuse. Other characteristics Victimisation, current/historical substance abuse.	4*
32. Girardi et al., (2009) (UK). Assessing the risk of inpatient violence in autism spectrum disorder.	Autistic service users admitted between 2014 and 2016 to St Andrew’s Healthcare, a low and medium security psychiatric hospital in England that provides specialist psychiatric secure and forensic care across several services.	Electronic records of 28 autistic male service users admitted to low and medium secure psychiatric hospital, including 12 non-violent service users. Age: M = 30.5, SD = 10.6. 16 violent service users. Age: M = 32.2, SD = 11.4.	Quantitative between groups study using a retrospective file review. Diagnosis made using the ICD-10, Autism Diagnostic Observation Schedule (Lord et al., 2001), the Autism Diagnostic Interview-Revised (Rutter et al., 2003), the Diagnostic Interview for Social and Communication Disorders (Wing et al., 2002).	Offence Assault, arson, sexual offence, attempted murder, threatening behaviour/threatening murder, possession of weapons. Psychopathology Psychosis, neurotic, stress, related and samatoform disorder, personality disorder, affective disorder, mental retardation, hyperkinetic disorder, stress related, mental retardation Other characteristics History of physical and verbal aggression	2*
33. Haw et al. ( 2013) (UK). Characteristics of male autistic spectrum patients in low security: are they different from non-autistic low secure patients?	Consecutive admissions between August 2008 and December 2012 of male service-users to two specialised low secure ASD units and one non-ASD unit at a tertiary referral centre (St Andrew’s Healthcare).	45 male autistic service-users recruited from two specialised low secure psychiatric services Age: 19–57 (Median = 27) Ethnicity: White British (n = 40), Other (n = 5) 43 control participants from a non-ASD unit. Age: 20–57 (Median = 33). Ethnicity: White British (n = 25), Other (n = 18)	Quantitative case-control study. Previous diagnosis confirmed using ICD-10.	Offence Violence, sexual offending, arson, stalking, damage to property, drug-related. Psychopathology Schizophrenia, alcohol abuse/dependence, depression, personality disorder, Mild learning disability, Hyperkinetic disorder, other diagnoses. Other characteristics History of childhood neglect/abuse, no qualifications, unemployed, history of antisocial behaviour/ convictions before the age of 18, single, sexually inappropriate behaviour, physical violence, history of self-harm	4*
34. Helvershou et al., (2018) (Norway). Personal experiences of the Criminal Justice System by individuals with autism spectrum disorders.	Autistic offenders in prison who had undergone a forensic psychiatric examination in Norway between 2000 and 2010. Sub-sample (n = 8) of Helverschou et al’s (2015**) **study, plus 1 additional participant.	9 autistic adults in prison (8 men and 1 women) Age: 21–50 years (M age = 34, SD = 9.5)	Qualitative: Semi-structured interviews. Diagnosis made using ICD-10 or previous mental health services.	Offence Arson, violence, murder, sexual offences, fraud and drink-driving. Psychopathology Intellectual disabilities, comorbid psychiatric disorder (personality disorder, affective disorders, substance abuse disorder). Other characteristics Themes included understanding of the crime, ways of preventing the crime, response to the arrest, the trial, coping and prison activities, interactions with prisoners, and life after the offence.	4*
35. Lundstrom et al. (2014) (Sweden). Childhood neurodevelopmental disorders and violent criminality: A sibling control study.	Population based study using several registers.	3391 Children born between 1984 and 1994 with neurodevelopmental disorders, including 954 with ASD.	Quantitative between groups longitudinal study. ICD 8, 9 & 10, DSM-IV (APA, 2000), Autism-Tics, ADHD, and other Comorbidities Inventory (A-TAC; Hansson et al., 2005; Larson et al., 2010).	Offence Violent crime including homicide, assault, robbery, arson, any sexual offence, illegal threats, intimidation. Psychopathology Mental retardation, ADHD, tic Disorder, obsessive compulsive disorder	5*
36. Murphy (2014) (UK). Self-reported anger among individuals with an autism spectrum disorder detained in high security psychiatric care: Do preoccupations have an influence?	High secure psychiatric hospital for assessment between January 2002 and December 2011.	20 Autistic male service users, including preoccupied offending group (n = 10) and non-preoccupied offending group (n = 10). Age: (M age = 34.15, SD = 13.2)	Quantitative between groups study. The diagnosis of autism was made/confirmed using the Autism Spectrum Quotient (Baron-Cohen et al., 2001), Adult Asperger’s Assessment (Baron-Cohen et al., 2005), the Diagnostic Interview for Social and communication disorders (Wing et al., 2002) and module four from the Autism Diagnostic Observation Schedule (Lord et al., 2000).	Offence Threats to kill, manslaughter, possessing explosives, murder, attempting to cause an explosion, rape, arson with intent to endanger life, manslaughter and indecent assault, wounding with intent, grievous bodily harm with intent, actual bodily harm, false imprisonment and wounding with intent. Psychopathology No individual had a full-scale IQ of 70 or below. Psychiatric comorbidity, mainly psychosis and personality disorder.	4*
37. O’Donoghue et al. (2014) (UK). Characteristics of referrals and admissions to a medium secure ASD unit.	A cohort of service-users referred to a specialist forensic medium-secure autism spectrum disorder service during its first two years of opening.	23 Males admitted. Age: (M age = 28.94, SD = 12.34). Ethnicity: 91.3% White Caucasian, 4.3% Black, and 4.3% Asian. 17 males not admitted Age: (M = 27.48, SD = 8.64) Ethnicity: 82.4% White Caucasian, 5.9% Black, 5.9% Mixed, and 5.9% Asian.	Quantitative between groups study using retrospective file review. Diagnosis of autism, although structured diagnostic tools for autism used in a small minority of cases (30%).	Offence Sexual offences, violent offences, and property offences. Psychopathology Learning disability, obsessive compulsive disorder, schizophrenia, anti-social personality disorder, bipolar disorder, ADHD, anxiety disorder, schizoaffective disorder, depression, and post-traumatic stress disorder. Other characteristics History of self-harm, no qualifications, no previous employment.	3*
38. Payne et al. (2020a) (UK). Self-reported motivations for offending by autistic sexual offenders.	Autistic offenders from four prisons and two probation services in England and Wales.	9 Male autistic offenders Age: 22–50 years (M = 29.56, SD = 8.68).	Qualitative semi-structured interviews. Participants were identified by criminal justice system (CJS) staff as being diagnosed with autism according to their records kept by the prison or probation service.	Offence Downloading and possession of indecent images, sexual assault, indecent assault, taking and distributing indecent images, causing, or inciting a child to engage in sexual activity and arranging and facilitating a child sex offence. Psychopathology Mean age at receiving an autism diagnosis was 13.13 years (SD = 4.90; range = 5.5–22 years). Other characteristics Social difficulties, misunderstanding, inadequate control, significant life changes, lack of support or altered mental state due to substances were factors in offending.	5*
39. Payne et al. (2020b) (UK). Are mental health, family and childhood adversity, substance use and conduct problems risk factors for offending in autism?	Males from an offending and non-offending population with and without ASD from England and Wales recruited from four prison establishments, two probation services, one approved premises and two secure hospitals. Autistic non-offenders were recruited via the National Autistic Society and the Research Autism website. Non offenders without ASD were recruited from recruitment agencies, local council facilities, and non-academic departments at the University of Bath.	40 Autistic male offenders. Age: M = 33.65, SD = 11.37. 40 autistic non-offenders. 40 non autistic offenders. 39 non autistic non offenders. (All males)	Quantitative between groups design. Identified as having a formal autism diagnosis made by a qualified professional from Criminal Justice System staff using the Autism Spectrum Quotient-10 (Allison et al., 2012).	Offence Violent offences, sexual offences, drug offences, driving offences, theft/burglary, public order offences, arson. Psychopathology Neurodevelopmental disorder (excluding autism), schizophrenia or other primary psychotic disorders, mood disorders, anxiety or fear related disorder, obsessive compulsive or related disorders, stress related disorder, personality disorders or related traits Other characteristics Family and childhood adversity risk factors, and conduct problems risk factors.	5*
40. Smith (2021) (UK). Causes and consequences of delayed diagnosis of autism spectrum disorder in forensic practice: A case series.	Autistic adult males in forensic settings whose diagnosis of ASD was delayed.	3 Autistic adult males whose diagnosis of ASD was delayed.	Qualitative case studies. Autism diagnosed by a professional.	Offence Violence and sexual offending. Psychopathology Mild/borderline intellectual disability, antisocial personality disorder, a history of alcohol use, ADHD, schizoid antisocial and borderline personality disorder, self-harm, and anxiety. Other characteristics History of victimisation.	2*
41. Vinter et al. (2020) (UK). ‘People don’t like you when you’re different’: Exploring the prison experiences of autistic individuals.	Autistic men in a UK prison that houses individuals who are serving sentences for sexual convictions.	7 Autistic men in prison and convicted of a sexual offense. Age: 23–47 (M = 34.43).	Qualitative semi-structured interviews. Diagnosis of autism confirmed by intellectual and developmental disability nurse and patient records.	Offence Sexual offence. Other characteristics Problematic social interactions, frustration, stress, agitation, and late diagnosis of Autism.	5*
42. White et al. (2017) (USA). Autism spectrum disorder and violence: Threat assessment issues.	Autistic male offenders with a history of violence.	5 Autistic male offenders with a history of violence.	Qualitative case study design. Diagnosis of autism using the DSM-IV (APA, 2000).	Offence Sexual assault and Murder. Psychopathology Dependent personality disorder, anxiety, severe obsessive-compulsive disorder.	2*
43. Crane et al. (2016) (UK). Experiences of autism spectrum disorder and policing in England and Wales: Surveying police and the autism community.	The police and autistic community online. Maras et al. (2017) recruited from the same sample.	31 autistic adults (16 males and 15 females). Age: 18–64. 49 parents of 33 autistic children (92% of parents had a male child and 70% had a female child) 394 police officers.	Mixed methods survey. Participants required to confirm that a clinical diagnosis of autism had been received.	Psychopathology Mood disorders, and anxiety disorders Other characteristics History of victimisation or discriminated against by police officers. 50% of respondents were in paid/voluntary employment and 2% were in education.	4*
44. Maras et al. (2017) (UK). Brief report: Autism in the Courtroom: Experiences of legal professionals and the autism community.	Judges, barristers, solicitor in criminal courts in England and Wales, and the autistic community. Sub-sample of autistic adults and parents/ carers as reported by Crane et al. (2016).	9 Autistic adults (8 males, 1 female). 19 parents/carers of autistic children (18 females, 1 male, reporting on 17 males and 2 females). 33 legal professionals.	Mixed methods survey. Self or parent/carer reported formal diagnosis of autism.	Psychopathology 79% had additional diagnosis. Other characteristics Age at diagnosis of Autism, 68% attended a mainstream school, 21% a special needs school, 7% a specialist unit in a mainstream school, and 1% home school. 11% had no qualifications. 44% not employed	4*
45. Brewer et al. (2016) (UK). Fitness to plead: The impact of autism spectrum disorder.	Autistic adults facing a criminal charge trial recruited from two independent secure hospitals in South-East England. Non-autistic adults recruited through advertisements in local news and community media in South East London.	Autistic males (n = 15) Age: 19–48 years (M = 27.53, SD = 7.81) A matched control group with no ASD diagnosis (n = 15), including 4 males and 11 females. Ethnicity: 8 White, 7 Black.	Quantitative between groups study. Autism previously diagnosed.	Other characteristics 14 of the autistic group had attended court previously, including 13 who had attended as a defendant, 2 who had attended as a supporter for a witness or defendant, and 3 who had sat in the public gallery during a trial.	4*
46. Gomes et al. (2014) (Portugal). Alleged biological father incest: A forensic approach.	Forensic medical examination reports and respective legal outcomes related to alleged cases of paternal incest of victims less than 18 years old in Portugal between 2003 and 2008.	215 Victims of alleged abuse, 1 of whom had ASD.	Quantitative cohort study using a retrospective file review. Formal diagnosis of autism reported in case file.	Other characteristics 1 victim had autism diagnosis (10-year-old female).	3*
47. Weiss & Fardella 2018 (Canada). Victimization and perpetration experiences of adults with autism.	Autistic and non-autistic adult victims and perpetrators in the community.	45 Autistic adults Age: 18–52 years (M = 30, SD = 1.48) 43.5% Men 15.6% self-identified minority status IQ: 81–134 (M = 110.22, SD = 13.9) 42 non autistic adults without intellectual disabilities.	Quantitative survey with a between groups design. Have a diagnosis of autism according to self-report, which was verified by administering the ADOS-II (Lord et al., 2012).	Other characteristics Vulnerability to violence victimisation during childhood, emotion regulation difficulties.	3*

Regarding overlapping samples, study 2 used the same sample of 269 violent offenders as both study 9 and 20. Study 7 used the same population sample of 3382 offenders as Study 15. Furthermore, study 34 used a sub-sample (n = 8) of participants recruited to study 7. Study 27 and 28 used the same sample of 35 autistic adults in two papers. Lastly, study 44 used a sub-sample of autistic adults (n = 9) and parents/carers (n = 19) as recruited to study 43. Papers with duplicate samples were included within the review as each reported unique findings. However, when calculating the total number of participants across studies, papers which made use of a previously reported sample were not included, to avoid inflating the sample size.

### Quality Assessment

Whilst the King and Murphy review did not use a specific quality appraisal tool, best practice guidelines suggest one is used, therefore, the Mixed Methods Appraisal Tool (MMAT) (Hong et al., 2018, 2019) was used for the current review. The MMAT is a 21-item checklist, used to rate the quality of quantitative (including cohort studies, cross sectional studies, longitudinal studies, case control studies and descriptive studies), qualitative, and mixed methods studies selected for review. Each article was appraised and scored using the criteria provided in the MMAT user guide (2018) by the first author and 20.8% were rated by the third author to assess reliability. Differences in ratings were found within five papers due to variation in interpretations for two criteria of the MMAT. Following discussions between the current authors, agreement on interpretation of criteria was reached resulting in complete agreement of ratings (inter-rater reliability: 100%). Studies were appraised based on five areas relating to the appropriateness of methodology, data analysis techniques and data collection techniques, the representativeness of the sample, reliability of outcome data, and the researchers’ interpretation of research findings.

## Results

### Quality Appraisal

Studies using only quantitative methods accounted for 36 of the 47 papers (1–11, 13–26, 29–30, 32, 35–37, 39, 45–47). Additionally, six qualitative papers were reviewed (12, 34, 38, 40, 41, 42), and five used mixed methods analyses, in which authors collected both qualitative and quantitative data predominantly from surveys (27, 28, 31 43, 44) and interviews (31). Of the 47 papers, 8 were rated as 5* (17%), 25 were rated as 4* (53%), 9 were rated 3* (19%), and 5 were rated as 2* (11%).

The quality of included papers was compromised by several methodological limitations (e.g., poor generalisability, unrepresentative samples, lack of matched comparison samples, reliance on retrospective data collection, lack of standardized instruments), which impacted study outcomes and limited the extent to which findings could be relied upon. Several authors of studies that took a qualitative approach to the research reported data collection methods that were considered inadequate (e.g., 27, 28 & 40), and/or did not support their interpretation of findings with sufficient data (e.g., 34, 40, & 42), which led to an unclear link between qualitative data sources, data collection, analysis, and interpretation (e.g., 40 & 42). In addition, several authors of quantitative studies with non-randomised comparison samples did not recruit a representative sample of participants (e.g., 1, 4, 22, 29, 30–33, 36, 45, 47). Due to an overreliance on retrospective data collection methodologies and database/documentary analyses, missing data was a consistent weakness of the included studies (e.g., 4, 18 19, 24, 32, 46). Moreover, confounding variables were rarely accounted for in the design or analysis of findings (e.g., 1, 2, 4–10, 13–17, 21, 29, 30–32, 37, 46). Only one study did include a matched comparison sample controlling for confounding variables (24). Altogether, authors recruited 606 autistic adults, 1,271 adults with intellectual disabilities and autism and a population control group of 2,973 young adults (24). Those studies that utilised a mixed methods design, did not meet the criteria for both of the different components of the methods involved.

### Study Characteristics

Of the 47 studies, 19 were conducted in the UK, 6 in Sweden, 6 in the USA, 4 in the Netherlands, 3 in Norway, 4 in Canada, 2 in Portugal and 3 in Australia. Of the 47 studies, authors of 9 recruited autistic people in contact with the CJS from state/nationally representative populations (14, 16, 22, 24–28 & 35). Authors of 13 studies recruited autistic people in contact with the CJS from psychiatric inpatient services (1, 4–6, 8, 11, 30, 32, 33, 36, 37, 40 & 45), 9 from prison and/or probation services (2, 9, 12, 17, 20, 23, 34, 38 & 41), 5 from the community (29, 31, 43, 44 & 47), 4 from forensic psychiatric examinations (7, 15, 42 & 46), 2 from police services (13 & 21), 2 from a residential treatment facility (18 & 19), 1 from a forensic outpatient service (3), 1 from community and inpatient forensic services (10), and 1 from a combination of prison, probation, and psychiatric inpatient services (39).

Of the 47 studies, authors of 20 recruited males only (2, 3, 9, 11, 12, 17–20, 23, 32, 33, 36–42 & 46), while authors of one study did not report the gender of participants (22), and authors of the remaining 26 studies recruited both males and females (1, 4–8, 10, 13–16, 21, 24–31, 34, 35, 43–45, 47).

Of the 47 studies, authors of 13 reported the age of autistic offenders (n = 326) ranging from 19 to 57.3 years (M = 31.50, SD = 11.03). Of the 47 studies, authors of 23 reported the gender of autistic offenders (n = 450), and most participants were male (n = 433) rather than female (n = 17). Findings are therefore biased towards autistic males.

Of the 47 studies, authors of only three studies reported the ethnicity of autistic offenders (n = 83). Ethnicities reported were White (n = 69), Black (n = 8), Asian (n = 1), and other (n = 5).

### Prevalence of Autism in the CJS

If autistic people were as likely as anyone else to be involved in the CJS we would expect the rates of autistic people in CJS populations to be about the same as the prevalence of autism in the general population (i.e., of the order of 1%).

Of the 47 studies included in the review, authors of 24 included prevalence data for autism amongst a sample of suspects or offenders (studies 1–24). A minority of studies reported using gold standard methods for diagnosing autism (2, 9, 11, 20), such as the ADI-R (Lord et al., 1994), Diagnostic Interview for Social & Communication disorders (DISCO; Wing et al., 2002) and the Autism Diagnostic Observation Schedule (ADOS; Lord et al., 2000; 2012). Several other studies used validated screening methods (17 and 23), including the Autism Spectrum Quotient (ASQ; Baron-Cohen et al., 2001), the Asperger’s Syndrome Diagnostic Scale (Myles et al., 2001). Other studies referred only to the ICD-10 or DSM-IV, perhaps due to a heavy reliance on retrospective case file data (e.g., 1, 4, 6–8).

Unbiased samples were recruited to a minority of studies (5, 7, 9, 12, 14, 15, 22). Most other studies recruited participants already hospitalised in psychiatric inpatient services who were therefore already identified as being a risk to either themselves or the public and as having a mental health condition (e.g., 1, 4, 6, 8 & 11). One study that recruited participants from the community, was also limited to people known to an outpatient psychiatric service (3). Even when unbiased samples were recruited, the methodological quality of these studies was limited as it was unclear how a diagnosis of autism was made and by whom (e.g., 5 & 7), or because samples of participants were limited by gender (e.g., 9 and 23), or type of offending behaviour (e.g., animal abuse or sexual offending; 19, 21).

Consequently, prevalence of autism amongst offending populations varied widely from 0.2% in a sample of offenders detained across seven prisons in New South Wales, Australia (12) to 62.8% in a sample of adolescent male offenders aged between 15 and 20 years who had been ordered by Juvenile Court into an adolescent sexual offenders’ program in the USA (19). Of the 16 studies to include prevalence data of autism derived from biased samples of offenders (n = 2926), 13.3% of offenders were reported to be autistic (n = 388; see Table 2). Of seven studies to include prevalence data of autism derived from mostly unbiased samples of offenders (n = 15,963), 3% of offenders were reported to be autistic (n = 486; see Table 2), though, none of the samples recruited can be said to be truly unbiased due to several factors, including the likelihood of prisons providing lower prevalence of autism due to people being diverted away from custody, as well as difficulties associated with the accuracy of diagnosis. The current prevalence of autism in offender populations is therefore slightly higher than the prevalence of autism in community samples, which range between 0.7 and 1.1% (Baxter et al., 2015; Brugha et al., 2016). Therefore, findings suggested autistic people may be somewhat over-represented in the Criminal Justice System, though biased samples and methodological limitations of the research conducted impact the reliability of these findings, for example, people recruited from the forensic psychiatric inpatient services often produce high prevalence rates as those referred are highly likely to have other mental health needs (King & Murphy, 2014). Moreover, it may be that suspects with limitations to their social behaviour (for example, poor eye contact) may be more likely to be seen as risky and therefore they may be more likely to receive custodial sentences. An added difficulty is that, due to varying samples and inconsistency in diagnosis assessment tools used when recruiting autistic suspects and offenders, it is difficult to make comparisons across studies.

### Autistic People and their Contact with the CJS

Offending is known to be generally more common amongst teenage boys, rather than older men and girls (Farrington et al., 2021). If autistic people are as likely to offend as other people without autism, we would expect to see this reflected in figures for comparison samples of autistic and non-autistic people, matched for age and gender.

Of the 47 studies, authors of five reported on the prevalence of autistic people’s involvement in the CJS. Here it is essential for studies to have a comparison group of non-autistic people involved in the CJS for any conclusions to be drawn. None of the included studies reported a robust procedure for diagnosing autism, but instead relied on previous diagnosis, which on occasions was self-reported (e.g., 27 & 28). Study 29 did however, verify a self-reported diagnosis using the Social Communication Questionnaire (SCQ; Rutter et al., 2003). Studies reporting on the prevalence of involvement in the CJS for populations of autistic people were therefore of limited methodological quality due to a lack of robust assessment of autism.

Consequently, prevalence of involvement in the CJS for populations of autistic people varied widely from 0.7% in a community sample of 284 autistic adolescents and adults in Ontario, Canada (29) to 5% in a nationally representative sample of 920 autistic youth aged 15–17 years in the USA (26). Overall prevalence of involvement in the CJS across the six studies (n = 6978) was 4.3% (n = 300; see Table 2). However, what constitutes involvement in the CJS varied between studies, for example, study 26 reported on those who had been arrested, whereas study 25 reported on those convicted of at least one violent offence. Consequently, how authors define involvement in the CJS will impact on prevalence data, whereby a broader definition including any contact with the criminal justice system will increase prevalence figures as those who are arrested or questioned by the police may not go on to be convicted. A lack of research including unbiased comparison samples limits our understanding of whether those who were autistic were at more or less risk of offending in comparison to non-autistic people. Nevertheless, other studies of non-autistic adolescents (e.g., Farrington et al., 1975; 2021) suggest that these figures for autistic adolescents are much lower than for non-autistic adolescents. As reported by Farrington & Hawkins (1991), by the age of 21 years, 31% of males from working class backgrounds in England (n = 411) had a criminal conviction.

### Types of Offences Committed by Autistic People

As can be seen in Table 2, types of offences committed by autistic individuals varied and included arson, property crime, sexual offending, drug offences, traffic incident/driving offences, stalking, fraud, theft, and robbery (e.g., 4, 7, 30, 31, 33, 34). Of the 47 studies included in the review, authors of 10 studies recruited a group of autistic people, as well as a comparison group of non-autistic people (1, 9, 10, 14, 24, 30, 39, 45, 47).

Authors of study 24 compared 45 autistic service-users recruited from two specialised low secure psychiatric services to 43 participants from a non-specialised low secure unit and reported autistic service-users were more likely to be younger, less likely to have a diagnosis of drug or alcohol misuse/dependence or personality disorder. Furthermore, autistic service users, when compared to non-autistic service users were less likely to have a lifetime history of physical violence or sexually inappropriate behaviour towards others or to be non-compliant with prescribed medication. They were also less likely to have a forensic history and their number of previous convictions tended to be lower (24). These findings are supported by study 10, in which findings suggested autistic people referred to forensic intellectual disability services had a lower rate of being charged with a criminal offence.

Limited research has been conducted to explore risk factors for autistic and non-autistic people for different types of offending. Authors of study 14 compared 143 autistic and 572 non-autistic juveniles with intellectual disabilities and other special educational needs involved in the CJS and reported autistic offenders were least likely to commit property offences. However, study 9 reported that at index conviction, autistic offenders were overrepresented in child sexual offending, when compared to non-autistic offenders. Though non-autistic offenders had more previous convictions (e.g., for drug related crimes), no difference in total number of prosecuted crimes was reported, although, the reliability of these findings is limited due to the sample consisting only of young male offenders detained within a prison establishment and convicted of violent offences only. Nevertheless, study 39 compared 40 autistic male offenders and 40 non-autistic offenders to a community sample of 40 autistic and 39 non-autistic people without a history of offending behaviour. Findings suggested autistic offenders scored higher for risk factors related to mental health, conduct problems, and family/childhood adversity (39).

In comparison, authors of study 30 compared autistic adults (n = 606), adults with intellectual disabilities (n = 1271), and population controls (n = 2973) and reported few group differences. Findings suggested that young autistic adults were not overrepresented in the CJS compared to their non-autistic peers and were less likely to be involved in the adult justice system than their peers. These findings are supported by the findings of study 47 in which similar rates across all forms of perpetration for autistic and non-autistic adults were reported.

Few studies compared the offending behaviour of autistic and non-autistic offenders to identify unique characteristics or similarities between these populations. Similarly, few studies compared autistic offenders to autistic people without an offending history to identify unique characteristics or risk factors amongst populations. Despite conflicting evidence regarding the similarities and differences between autistic and non-autistic offenders, authors of study 45 compared autistic males (n = 15) to a matched control group of non-autistic males (n = 15) on their ability to understand and follow court proceedings and stand trial. Findings suggested that autistic adults had more difficulty understanding and following aspects of the trial process and proceedings, despite their prior experience of the courtroom process. These findings suggest autistic adults who encounter the CJS have unique needs.

### Co-morbid Psychiatric Diagnosis

Authors of 33 of the 47 studies reported on comorbid psychiatric diagnosis of autistic offenders (see Table 2). Most authors recruited samples who were already in a mental health hospital or secure state facility (4, 11, 17 19, 30, 32, 33, 36 & 37), or who had been referred for forensic psychiatric assessment (7, 10, 15 & 34). Additionally, authors of five studies recruited from prison and probation services (study 2, 9, 21, 23, 38), while authors of one study recruited from the prison, probation, one approved premise and two hospitals (39), and authors of six studies included recruited participants from community samples (study 27, 28, 29, 31, 43, 44). However, in studies 32, 46 and 47, participants comprised autistic individuals who had interacted with the police for a variety of reasons (e.g., victimisation, mental health crisis) and therefore not all participants had a history of offending behaviour. Furthermore, two studies of poor methodological quality failed to report the type of forensic services in which autistic offenders were placed (40 & 42). Common psychiatric diagnoses reported included attention deficit hyperactivity disorder (ADHD), Tourette syndrome, intellectual disabilities, substance related disorders, psychosis, personality disorders, anxiety, mood disorders, and obsessive-compulsive disorder. Study 4 reported the prevalence of comorbid diagnosis of substance misuse and personality disorder were significantly lower in the autistic group compared to the non-autistic group. Study 33 reported the age at first contact with psychiatric services for the autistic group was lower compared to the non-autistic group, but age at first psychiatric admission was not significantly different. Further, 73.3% had psychiatric comorbidity, most commonly schizophrenia, but unlike controls, personality disorder and drug and alcohol disorders were uncommon. Additionally, a lifetime history of drug use, but not alcohol use was significantly less common for the autistic group, when compared to the non-autistic group. Study 39 reported that autistic offenders scored higher on the mental health risk factors than both typically developing offenders and typically developing non-offenders. Study 16 reported that serial offenders were more likely to have a personality disorder or autism when compared to single offenders.

Authors of five studies used a population-based cohort leading to findings that are likely to be more representative of offenders with an autism diagnosis (20, 22, 25, 26, 35). These studies showed that co-occurring intellectual disabilities and ADHD were most common, as well as psychotic disorder, drug and alcohol misuse, personality disorder, affective disorders, anxiety, tic disorder, and obsessive-compulsive disorder. Authors of study 25 compared autistic and non-autistic individuals on the Swedish national crime register and found autistic individuals who also had ADHD and/or conduct disorder had an increased risk of violent criminality. Furthermore, autistic individuals with intellectual disabilities were less likely to be convicted, and autistic individuals without intellectual disability were more likely to be convicted, compared to people without any of these conditions. However, other methodological issues affecting the reliability and validity of findings include retrospective data collection, a lack of robust assessment, and female offenders being underrepresented.

### Experiences of the CJS

Several studies recruited participants from the autistic community, asking them about their experiences of the police in England, Wales, and Australia (31, 43, 44). Findings suggested that over 60% of respondents were dissatisfied with their experience with the police (31 & 43). The autistic community reported a lack of police awareness and knowledge of autism, a lack of information and explanation given by the police, and delays throughout the CJS (43). Furthermore, the needs of autistic individuals were often not met, and some individuals felt victimised or discriminated against by police officers (43). Interestingly, some researchers have explored whether autistic people disclose their diagnosis to the police, with findings suggesting as many as 36% never disclosed and 25% only disclosed their diagnosis on some occasions (study 43). Furthermore, their decision to not disclose was linked to a fear of discrimination or victimisation by police officers (study 43). Studies 27 and 28 recruited 35 autistic adults, 80% of whom reported to have interacted with the police at least once in their lifetime (n = 29), including being stopped by police (n = 17), being a victim of a crime (n = 17), for a mental health crisis (n = 11), suspected of a crime (n = 8), placed in handcuffs (n = 8) and convicted (n = 2). As many as 42.3% (n = 11) of respondents reported that the police had used force during the described interaction, including restraint (36.4%, n = 4), handcuffs (36.4%, n = 4), and assault (n = 3). Furthermore, none of the respondents indicated that the officer(s) were able to identify that the person with autism was vulnerable (27 & 28).

Other researchers have explored the experiences of autistic offenders in prison establishments (e.g., 12, 34 & 41). Research has highlighted the challenges surrounding the misunderstanding of autism in relation to social interactions with staff and other prisoners, which on occasions has escalated into altercations. Difficulties coping with feelings of frustration, stress, fear, upset, confusion, anxiety, and agitation that were caused by the unpredictability of the prison environment (i.e., changes in prison routine), and noise was reported as very difficult to cope with, as well as a lack of staff understanding regarding their diagnosis of autism. In contrast, some autistic offenders (n = 6) have reported coping in prison, as it provided structure and opportunities for work and study (34). It is likely that prisons vary in their ability and willingness to support autistic offenders. For example, Her Majesty’s Young Offender Institution Feltham, Her Majesty’s Prison Parc and Her Majesty’s Prison Wakefield have received autism accreditation from the National Autistic Society demonstrating their commitment to providing support to autistic offenders (Hughes, 2019; Lewis et al., 2015).

### Risk Factors for Offending

Several studies explored other risk factors associated with autistic offenders, including being male (29), single (7, 33), not having children (33), having birth defects (10), behavioural issues during childhood (14), poor educational attainment (7, 37), lack of regular employment (7, 37), poor social networks (7), having a history of verbal and physical aggression (4, 29, 32), offending/antisocial behaviour (33, 45), sexually inappropriate behaviour (33), substance misuse (31, 38), and a history of victimisation (4, 10, 19, 26, 27, 28, 31 33, 40 & 46). A range of parental and familial characteristics, such as belonging to households with lower incomes, migrant households, parental history of criminal convictions, and maternal psychiatric disorders were associated with violent criminality in autistic individuals (40). Authors of study 7 reported that 29% of the group had been in foster or institutional care as children (n = 14) and 4% had more than three changes of (non-family) carer during childhood (n = 2). Furthermore, a delayed diagnosis of autism has also been identified as a potential risk factor for offending (15, 25, 40). Several studies also reported on the motivations of autistic offenders. Common motivations included revenge, social misunderstanding, idiosyncratic rationalisations or explanations, obsessions/special interests, and social naivete (7, 15, 38).

However, autistic offenders were rarely compared to non-autistic offenders in these analyses, meaning their unique characteristics have not been identified. Nevertheless, authors of study 7 reported on a sample of 48 autistic offenders who had undergone forensic examination between 2000 and 2010 in Norway and found that 56% had no previous convictions (n = 27). Furthermore, authors of study 33 reported that a lifetime history of sexually inappropriate behaviour (n = 13) and physical violence (n = 35) were less common for the autistic offenders, when compared to non-autistic offenders. Similarly, study 9 recruited 269 violent male offenders, including 26 autistic offenders. Findings suggested there were few differences between autistic and non-autistic offenders, including prevalence of psychopathy, although, childhood placements in foster homes were overrepresented in the autistic group. Study 39 recruited arguably a more representative sample of males from an offending and non-offending population with and without autism from England and Wales from four prison establishments, two probation services, one approved premise, two secure hospitals and the community. Autistic offenders reported higher scores on the offending risk factors questionnaire (unpublished) than non-offenders and also offenders without autism. Autistic offenders scored significantly higher than the non-autistic, non-offender group on family and childhood adversity risk factors. Autistic offenders scored higher than both the autistic non-offenders and non-autistic non-offenders on the conduct problems risk factors. However, few other studies have sought to compare findings between populations, and due to biased sampling, the findings of the current review may be an overestimation of aggression and violence in autistic offenders.

Furthermore, autistic people who encounter the CJS also appeared to be vulnerable to social isolation and segregation following arrest, due to difficulties associated with interpersonal skills and communication (11, 12). For example, study 26 reported that among a nationally representative sample of 920 autistic youth, 27.5% had experienced social isolation. In addition, research also suggested as many as 97.6% of autistic offenders having a history of self-harm (n = 41; study 4 & 37). Furthermore, the prevalence of self-harm in the autistic group was significantly higher than the non-autistic group (4). In contrast, study 33 found that a history of self-harm was less prevalent for autistic service-users admitted to a secure psychiatric inpatient service, compared to non-autistic service-users (n = 17), although the difference did not reach statistical significance.

### A History of Victimisation

Authors of several studies identified that autistic people who encounter the CJS often have a previous history of victimisation. For example, study 4 recruited 138 service-users from a specialised forensic inpatient intellectual disabilities service and findings suggested 40.5% of autistic service-users reported being a victim of any type of abuse (n = 17), and 33.3% reported being a victim of sexual abuse (n = 14). Similarly, study 10 reported on referrals made to forensic community and secure intellectual disability services in England and Scotland and found 23.4% of autistic people had been abused as a child (n = 11). In contrast, authors of study 33 recruited a control group and compared autistic and non-autistic service users admitted to two secure psychiatric inpatient services and found a lower percentage of the autistic group had a history of childhood neglect or abuse. However, study 19 which recruited 43 male adolescents adjudicated ‘delinquent’ due to a sexual offence in a state residential facility in Pennsylvania, reported emotional abuse and neglect were more common in the autistic group compared to the non-autistic group.

## Discussion

### Summary of Findings and Interpretation

Findings of the current review suggested a limited amount of good quality research has been conducted that has focused on improving our understanding of autistic people in contact with the CJS since 2013. Findings suggested that as with the general population, autistic people engage in a range of offending behaviours. However, studies of autistic people who encounter the CJS are still in their infancy and our understanding of their unique characteristics and treatment needs remains limited. Much like the studies included in the previous review by King and Murphy, most of the findings from the updated literature have originated from biased samples (i.e., mental health services) and are unlikely to reflect all autistic people who may be in contact with the CJS (e.g., individuals cautioned, arrested, fined, living in the community, those convicted of minor offences, or those in custody awaiting trials). As with the previous research identified by King & Murphy (2014), study methodologies made comparisons regarding prevalence across studies difficult, particularly due to a lack of matched control groups. Limitations of the research impact on the reliability of current findings, which should therefore be interpreted with some caution.

King & Murphy (2014) reported the prevalence of autism amongst offender groups varied between 1 and 27%. Similarly, in the current review prevalence did vary considerably depending on study methodology and was limited by the use of biased samples recruited from prisons and secure mental health services. These limitations notwithstanding, the prevalence of autism in offending populations was estimated to be between 0.2 and 62.8% (studies 12 & 19).

In relation to the rather different question of the prevalence of offending within samples of autistic people, King & Murphy (2014) reported figures varied widely between 2.74 and 26%, although when reviewing studies that included a control group, results suggested autistic people committed the same number of offences or fewer offences than non-autistic people. In the current review, the prevalence of offending obtained from six samples of autistic people, was estimated to be between 0.7 and 5.7% (see Table 2). In England and Wales, the prison population as of March 2022 was 79,808 suggesting a prevalence of 0.1% (Ministry of Justice, 2022), although these figures underrepresent the number of people to encounter the CJS as suspects or offenders, excluding individuals in the community. Therefore, as concluded by King & Murphy (2014), when these rates are compared to the rates of offending amongst non-autistic people, the current evidence suggested autistic people may be no more likely than non-autistic people to engage in offending behaviour and encounter the CJS, although these findings should be interpreted with caution, given the limitations of the evidence base (i.e., unrepresentative, and biased samples, varying definitions of offending behaviour). Further, the lack of research on autistic females engaged in the CJS warrants further exploration. Such a small sample of autistic females recruited to some studies may, in part, provide some explanation for the variation in prevalence data. However, evidence continued to suggest autistic offenders engaged in a variety of offending behaviours, though the research remained biased in its focus on male autistic offenders and on violent and sexual offending with little consistency as to whether autistic people are more at risk of committing these types of offences.

As previously reported by King & Murphy (2014), comorbid psychiatric diagnoses continued to be common amongst autistic offenders, in particular ADHD, intellectual disabilities, psychosis, personality disorder and other affective disorders (e.g., 25). However, researchers have continued to recruit biased samples, predominantly from forensic psychiatric inpatient services, thus re-iterating the need for research into the prevalence of poor mental health, learning disabilities and other vulnerabilities, including autism as emphasised seven years ago in a report published by the Centre for Mental Health (Durcan et al., 2014).

Findings of the current review suggested that many characteristics associated with offending behaviour for autistic people are similar to those who are not autistic, including socio-demographic background, parental history, history of abuse, poor education attainment, lack of regular employment, and a history of aggression (e.g., studies 4, 7, 10, 29, 32, 37, 40, 47). However, some new findings have emerged since King & Murphy (2014), which have suggested that autistic offenders do have some unique characteristics that require consideration in service development and in their care and treatment. New characteristics that emerged from the current findings included evidence to suggest autistic offenders are less likely to have previous convictions related to drug offences, although they will sometimes misuse substances (e.g., study 9). Whilst few studies focused on identifying the motivations of autistic people who offend, it is suggested that some appear to be unique to this group of individuals and warrant further exploration (e.g., social misunderstandings, idiosyncratic explanations, social naiveté, and circumscribed interests; studies 7, 15 & 38), as King and Murphy also found.

More significantly, the current review highlighted new characteristics of autistic people in contact with the CJS in that they appear vulnerable to victimisation and abuse (e.g., study 4, 47), and are at increased risk of self-isolation and self-harm (e.g., 4 & 37). Further, research exploring the experiences of autistic people who encounter the CJS suggested more work is needed to address the lack of knowledge and awareness of autism amongst professionals working within the CJS (i.e., the police and prison staff). Research pertaining to judges and the court process was specifically lacking, with no studies identified that focused on these areas of the CJS. Further training is required to ensure autistic people are identified and provided with the appropriate support at all stages of the CJS. It is notable that in the UK, where the National Autistic Society accredit prisons that they consider the vulnerabilities of autistic people and their unique needs (e.g., sensory issues), unfortunately, only three of the 118 prisons in England and Wales, have received accreditation, which raises concerns about the support autistic offenders could expect to receive in a prison environment (Hughes, 2019).

### Strengths and Limitations

The findings of the current review are limited by the relatively poor quality of included articles. Most studies used data retrieved from online questionnaires or file information from records and archives. Few articles collected primary data from autistic offenders. Although the current review sought to include global research on autistic offenders, the majority of research related to people in the UK and findings may therefore be unrepresentative of autistic people in other countries. Nevertheless, the current review has several strengths. Qualitative, quantitative, and mixed methods studies, were reviewed, highlighting several gaps in our understanding of autistic offenders. Unlike King & Murphy (2014), an objective measure of the assessment of quality of all included articles was also utilised.

### Implications for Policy, Practice, and Future Research

Since 2013, several policies and guidelines have emphasised the need to provide person-centred, and evidence-based support to autistic people who encounter the CJS in the UK and elsewhere. However, very little research has been conducted to evaluate their implementation. The current review suggested that further research needs to be conducted to provide a more accurate and reliable estimate of offending amongst autistic people. The few studies that focused on the experiences of autistic people in the CJS (e.g., 12, 31, 34) highlight the need for autism training for staff across the CJS, including police, prison staff, and judges. There is a need for increased recognition of autism, and increased support for autistic people in contact with CJS to improve care and treatment outcomes. Additionally, support needs to be in place for those with autism already in secure/custodial facilities to enable them to navigate the unpredictable environments and confusing social system within prison. In addition, more information and research on autistic people of other genders (e.g., female, non-binary) as well as ethnicities within the CJS are needed. Relevant strategies should be developed to ensure autistic people are identified and supported throughout all stages of the CJS. Professionals working within the criminal justice system should be trained on autism. Training should be evidence-based and empirically evaluated. The experiences of autistic people who encounter the CJS, as well as their carers should be sought and valued to ensure improvements to the care and treatment of autistic individuals in the CJS.

## Conclusions

The current evidence base remains fraught with methodological limitations, with research conducted often using small unrepresentative samples. Nevertheless, findings suggested that autistic people do encounter the CJS as offenders, in addition to sometimes being victims of crime. In addition, autistic people appear largely comparable to non-autistic people, in terms of risk factors like social deprivation, though there was some evidence to suggest there are some unique characteristics which require careful consideration in the context of forensic care and treatment. Several policies and guidelines have been introduced to improve the care and treatment outcomes of autistic people in contact with the CJS but their implementation and effectiveness have not been sufficiently evaluated. Evidence suggested that autistic people have a range of vulnerabilities and require additional support, which appeared to be lacking in services where the experiences of autistic people have been examined.
